# The Skin and Natural Cannabinoids–Topical and Transdermal Applications

**DOI:** 10.3390/ph16071049

**Published:** 2023-07-24

**Authors:** Silviu-Iulian Filipiuc, Anca-Narcisa Neagu, Cristina Mariana Uritu, Bogdan-Ionel Tamba, Leontina-Elena Filipiuc, Ivona Maria Tudorancea, Andreea Nicoleta Boca, Mădălina Florina Hâncu, Vlad Porumb, Walther Bild

**Affiliations:** 1 Advanced Research and Development Center for Experimental Medicine (CEMEX), Grigore T. Popa University of Medicine and Pharmacy, Universitatii Street, 16, 700115 Iasi, Romania; silviu.filipiuc@umfiasi.ro (S.-I.F.); cristina-mariana.uritu@umfiasi.ro (C.M.U.); leontina.filipiuc@umfiasi.ro (L.-E.F.); ivona-maria.scafariu-tudorancea@d.umfiasi.ro (I.M.T.); 2 Department of Physiology, Grigore T. Popa University of Medicine and Pharmacy, 16 Universitatii Street, 700115 Iasi, Romania; walther.bild@umfiasi.ro; 3 Laboratory of Animal Histology, Faculty of Biology, “Alexandru Ioan Cuza” University of Iasi, Carol I bvd, No. 20A, 700505 Iasi, Romania; aneagu@uaic.ro; 4 Department of Pharmacology, Clinical Pharmacology and Algesiology, Grigore T. Popa University of Medicine and Pharmacy, Universitatii Street, 16, 700115 Iasi, Romania; 5 Department of Pharmacology, Toxicology and Clinical Pharmacology, Iuliu Hatieganu University of Medicine and Pharmacy, 400347 Cluj-Napoca, Romania; boca.andreea@umfcluj.ro; 6 Arcadia Policlinic, 700083 Iași, Romania; madalinahancu92@yahoo.com; 7 Department Surgery, Grigore T. Popa University of Medicine and Pharmacy, Universitatii Street, 16, 700115 Iasi, Romania; vlad.porumb@umfiasi.ro; 8 Center of Biomedical Research of the Romanian Academy, 700506 Iasi, Romania

**Keywords:** skin endocannabinoid system, natural cannabinoids, phytocannabinoids, topical cannabinoids, transdermal administration, delivery systems, nano-formulations

## Abstract

The chemical constituents of the *Cannabis* plant known as cannabinoids have been extensively researched for their potential therapeutic benefits. The use of cannabinoids applied to the skin as a potential method for both skin-related benefits and systemic administration has attracted increasing interest in recent years. This review aims to present an overview of the most recent scientific research on cannabinoids used topically, including their potential advantages for treating a number of skin conditions like psoriasis, atopic dermatitis, and acne. Additionally, with a focus on the pharmacokinetics and security of this route of administration, we investigate the potential of the transdermal delivery of cannabinoids as a method of systemic administration. The review also discusses the restrictions and difficulties related to the application of cannabinoids on the skin, emphasizing the potential of topical cannabinoids as a promising route for both localized and systemic administration. More studies are required to fully comprehend the efficacy and safety of cannabinoids in various settings.

## 1. Introduction

For over two millennia, the *Cannabis* plant has been used for both recreational and therapeutic purposes [1,2], with various archaeological discoveries confirming that it has been cultivated across most of the known world. Ancient Chinese medicine, for example, used the plant for musculoskeletal conditions and to relax and harmonize the mind and body [3], while the Greeks and Romans used it for recreational consumption and medical use [4]. The plant’s psychoactive and analgesic qualities were used by Indians to treat various types of pain [5,6]. In the first *Chinese Pharmacopeia*, *Cannabis* seeds were recommended for the treatment of skin diseases and disorders, such as eczema, psoriasis, and other inflammatory conditions [7]. Nowadays, due to the increasing interest in sustainability and ecological issues, the use of plant-based natural products in dermatology has become a valuable method for improving therapies with respect to acute and chronic skin diseases and disorders, with lower costs for patients and healthcare systems [7]. *Cannabis*, a representative genus of the *Cannabaceae* family [8], is an annual herbaceous plant, which includes the *Cannabis sativa* L. (hemp and marijuana), *C. ruderalis* Janisch, and *C. indica* Lam. species [9]. *Cannabis* is a genus characterized by dioecy [10], producing male and female inflorescences on different individuals that are obligatory out-crossers [11]. The plant is naturally occurring and is part of the local flora in the Indian Peninsula and Central Asia; however, in the rest of the world, it is cultivated for a variety of purposes. Cannabis is used in its unprocessed state as dried flower bulbs, known as marijuana, and as pieces of resin, known as hashish [12].

What makes each variety or strain of the *Cannabis* plant unique in its own way is the presence of three different classes of chemical compounds that exhibit biological activity: phytocannabinoids (pCBs), flavonoids, and terpenes/terpenoids [13]; they may act either individually and/or synergistically [14]. Depending on the plant content in each of these molecules, various modulatory or potentiation pharmacological effects have been observed [15,16]. A recent classification of *Cannabis* plant constituents identified 545 entities divided into chemical classes based on their structural similarities [17]. Thus, more than 100 *Cannabis* constituents belong to the pCB class, which is mostly extracted from female plants, with the most well-known being tetrahydrocannabinol (THC) with its two derivatives Δ8-tetrahydrocannabinol (Δ8-THC) and Δ9-tetrahydrocannabinol (Δ9-THC); cannabidiol (CBD); and cannabigerol (CBG) [18,19]. The rest of the natural compounds are classified into seven other main classes: cannabinol (CBN), cannabielsoin (CBE), cannabichromene (CBC), cannabicyclol (CBL), cannabinodiol (CBND), cannabitriol (CBT), and other types of cannabinoids [20].

The pharmacokinetics and metabolism of natural cannabinoids depend on the route of administration [13]. pCBs are known to have high lipophilicity, low aqueous solubility, rapid metabolism, poor bioavailability, and erratic pharmacokinetics [21], being temperature-, light-, and auto-oxidation-sensitive [22]. For example, the oral bioavailability of CBD ranges between 13% and 19% [23] due to its incomplete oral absorption and high hepatic clearance [2], while its bioavailability increases at 34–46% when it is absorbed intranasally [24]. Also, after topical in vivo gel applications, CBD provided significant plasma levels [24]. Consequently, pCB formulations emphasize a low oral bioavailability that led to the necessity of other administration routes to improve pCBs’ bioavailability and efficacy, such as the local (topical), transcutaneous (transdermal), pulmonary, and transmucosal routes [25] that include intranasal and rectal routes, with the last one demonstrating double the rate of bioavailability compared to the oral route [22]. Topical and transdermal administration demonstrated several advantages, such as a higher bioavailability rate, prolonged steady-state plasma concentration, and a reduction in psychoactive impacts of the drug due to its passive diffusion in the main barrier of the skin, *stratum corneum* [22]. To evaluate the efficacy of pCB topical applications, a plethora of studies emphasizing the mechanisms of action at the molecular level, redox transformation, electronic structure, and stability; and the cytotoxic, phototoxic, and UVA or UVB photoprotective effects of pCBs have been performed [26]. To emphasize the mechanisms of action of pCBs at the molecular level, it has been demonstrated that CBD inhibits nuclear factor kappa B (NF-κB) and the genes involved in the expression of molecules with pro-inflammatory roles, such as cytokines and metalloproteinases [27]. Clinical studies emphasized that topical treatment with CBD-enriched ointment significantly improved skin parameters and disease symptomatology, proving that the topical application is a safe and non-invasive alternative for improving the quality of life in patients with various skin disorders [28].

Outside of clinical studies, to investigate the potential roles of pCBs for the treatment and alleviation of skin diseases and disorders, many types of cell lines have been used in various in vitro studies: inflamed human keratinocytes (HaCaT cells) [29], human SZ95 sebocytes [30], human eccrine-sweat-gland-derived immortalized NCL-SG3 model cells [31], human healthy and stress-induced premature senescent (SIPS) CCD-1064Sk dermal fibroblasts [32], or My-La and HuT-78 cutaneous T-cell lymphoma lines [33].

Skin conditions affect between 30% and 70% of people worldwide and more than 3000 acute and chronic skin diseases that significantly reduce the patients’ quality of life have been described [34]. Traditional systemic treatments used in dermatology are based on topical agents (corticosteroids, vitamin D analogues, moisturizers, retinoids, and calcineurin inhibitors), phototherapy, systemic immunotherapy, systemic antibiotics and hormones, antifungals, biological agents, or small molecule inhibitors. These classic treatments may emphasize several limitations, including poor responses to treatment, potential drug interactions, hepatic and renal toxicity, and even a significant risk of skin malignancy and teratogenicity [28]. The most significant adverse effects of current drug-based therapies applied in the most frequent skin diseases, such as psoriasis, atopic dermatitis, allergic contact dermatitis, asteatotic eczema, acne, and seborrhea, are listed in Table 1: xerosis; erythema; purpura; rosacea or alteration in skin pigmentation; photoaging; skin atrophy; itching; burning sensation; folliculitis; actinic keratosis; allergic reactions; hair loss or hypertrichosis; hyper-lipidaemia; hypertension; metabolic and nutritional disorders; headache; bone marrow suppression; leukopenia; bone growth stimulation; decreased bone density; the inhibition of the hypothalamic–pituitary–adrenal axis in children; herpes simplex reactivation; and delayed wound repair [35,36,37,38,39,40,41,42,43,44]. In dermatology, pCBs have demonstrated anti-inflammatory [27,30], anti-oxidative [45], anti-aging [32], anti-acne [30], and anti-UVA/UVB radiation damage proprieties [46]. Moreover, several pCBs emphasized potent antimicrobial activity against Gram-positive bacteria, including methicillin-resistant *Staphylococcus aureus* (MRSA) that may cause serious infection on the skin and other organs [47], which is possible due to the inhibition of *Staphylococci* adhesion to keratinocytes [48]. Interestingly, Luz-Veiga et al. have unveiled that CBD and CBG may be promising topical antimicrobial agents for different skin conditions without a negative impact on skin microbiota [48]. Feldman et al. have shown that CBD can be considered an alternative treatment against fungal infection due to its anti-biofilm activity against *Candida albicans* [49], which resides on the skin surface and may cause infections [50].

Consequently, *Cannabis* plant-derived compounds have been recommended for the treatment of many skin disorders and diseases, such as dry/seborrheic skin and acne [30]; atopic dermatitis (or eczema); psoriasis and resulting outcome scars [28,51]; pruritus, including neuropathic itch [52]; pain [53]; and mycosis fungoides, the most common type of cutaneous T-cell lymphoma (CTCL) [33]. An international cross-sectional survey study demonstrated that cannabinoid-based medicine (CBM) may alleviate pain and pruritus and improves wound healing and well-being in patients with epidermolysis bullosa (EB), a group of clinically and genetically heterogeneous genetic skin conditions that express fragile skin and mucosae [54]. In vivo studies demonstrated that, individually or combined, cannabinoids may reduce tumor growth or tumor cell proliferation and promote apoptosis and autophagy in melanoma cells, emerging as potential agents for the treatment of cutaneous melanoma [55]. Lichen simplex chronicus and multiple cutaneous squamous cell carcinoma (SCC) lesions have completely regressed with the topical application of 20% CBD oil [56]. Psoriasis, which emphasizes two clinical manifestations, psoriasis vulgaris and psoriatic arthritis, is the most common autoimmune skin disease that currently affects about 4% of the population [51]. pCBs are known as candidate drugs in the treatment of psoriasis due to their effects of inhibiting the proliferation of keratinocytes and modulating the associated inflammatory response [57]. Acne vulgaris (acne) affects 9.4% of the world’s population and 85% of adolescents [58]. CBD is well known for its anti-inflammatory effects on acne [58]. Both CBG and cannabigerovarin (CBGV) exert potential in the treatment of skin dryness, while CBC, cannabidivarin (CBDV), and Δ(9)-tetrahydrocannabivarin (THCV) have been proposed as novel anti-acne agents due to their efficacy [30]. In regions where cannabis use is legal, there are reported clinical cases where topical formulations including phytocannabinoids (CBD and THC), self-initiated by patients or prescribed by certain dermatologists and prepared by pharmacists, have been shown to be effective in treating a variety of skin conditions [59,60,61].

Cannabinoids work on the endocannabinoid system (ECS) [62]. The endocannabinoid receptors and their endogenous ligands were discovered and identified after the first compounds in the class were isolated and characterized. The endogenous endocannabinoid system (ECS) is represented by a molecular signaling network consisting of endocannabinoids, specific G-protein-coupled receptors (GPCRs), cannabinoid receptor 1 (CB1) and cannabinoid receptor 2 (CB2), and specific enzymes responsible for the synthesis, transport, and degradation of endocannabinoids [59]. Various studies have also identified non-CB1/CB2 receptors modulated by endocannabinoids, such as GPCRs (GPR18, GPR55, and GPR119), transient potential vanilloid receptors (TRPV1 and TRPV3), transient receptor potential ankyrin 1 (TRPA1), nuclear hormone receptors like peroxisome proliferator-activated receptors (PPARs), and others [2,63]. ECS plays a critical role in a number of physiological processes, including immune modulation, vasomotion and vasodilation, motor control, muscle fiber formation, cognitive memory, learning, anxiety, appetite, gastro-intestinal motility, sleep, fertility, lipogenesis, and the formation of insulin and muscle fibers, as well as intestinal and bronchial motility [15,64,65,66,67,68]. It also plays a key role in pathological processes like pain, inflammation, and cancer [69,70,71]. Minor pCBs, such as CBG, CBC, THCV, and cannabigerolic acid (CBGA), exert their anti-inflammatory effect in human keratinocytes via the molecular deregulation of endocannabinoid (ECB) signaling and the mitogen-activated protein kinase (MAPK) pathway [29].

Cannabinoids’ beneficial or harmful effects are determined by how they act on the CB receptors and by their affinity for a particular receptor. The cannabinoids can also interact with a multitude of other receptors, which explains the variety of effects encountered in these compounds [20]. Being fully represented in the skin, the ECS contributes significantly to the body’s homeostasis [59]. By controlling, among other things, cell growth, differentiation, and survival; immune and inflammatory responses; and sensory phenomena, the ECS is also involved in the physiology and pathology of skin functions [72].

The skin is an accessible organ for the non-invasive or minimally invasive administration of medications [73]. However, the variability in skin permeability remains a challenge in optimizing transdermal drug delivery that targets the epidermis, dermis, deeper tissues, and systemic circulation [74]. Hence, the skin can be approached from both points of view as a therapeutic target and as a route of systemic administration.

The skin has the advantage of being an easy and convenient route of administration for a wide range of active ingredients, including natural or synthetic cannabinoids. Various techniques for the enhancement of skin permeabilization are available for the improvement of bioavailability and the efficacy of therapeutic cannabinoids, such as the use of organosilane particles as transdermal delivery vehicles [75], oleic acid and ethanol as good chemical enhancers, ethosomes and nanocryogels as controlled release strategies, and microneedle arrays for both the topical and transdermal delivery of incorporated cannabinoids [76].

Complementary and alternative medicine (CM and CAM) therapies have an increasing presence in dermatology [77], gaining popularity among patients in recent years. Natural remedies are seen as safer than conventional medications, with few if any notable adverse effects. However, dermatologists are wary of prescribing cannabinoid-based remedies due to the lack of studies regarding the formulation and standardization of extracts and safety profiles [78]. There are a few reported clinical cases of allergies related to topical cannabis contact, such as pruritus, urticaria, or angioedema, which were confirmed via specific allergy tests [79]. Similar o many other natural products or extracts, cannabis-based medicines have the potential to cause skin allergies in some individuals. However, thanks to advances in formulation techniques, exposure to some cannabis plant constituents may be significantly reduced.

The current review aims to bridge the gap between cannabinoids as traditional healing herbal extracts and the current science regarding optimal usage, including the formulation, route of administration, dosage, and frequency of use of these compounds in dermatological disorders and diseases.

## 2. The Topical versus the Transdermal Route of Administration for Natural Cannabinoids

### 2.1. The Skin as a Potential Therapeutic Target

Skin diseases like atopic dermatitis (AD), allergic contact dermatitis (ACD), asteatotic eczema (AE), psoriasis, pruritus, varicose ulcers, wounds, acne, and seborrhea are challenging to treat, have a significant impact on patients’ quality of life, and assume high costs in the long term throughout the entire life. With annual costs of over EUR 30 billion per year in Europe and USD 5.3 billion in the USA and treatments that are trying to modulate the microbiome or innate immune response, targeting itching or inhibiting Janus kinases, AD affects up to 20% of children and up to 10% of adults worldwide [80]. This pathology significantly reduces the quality of life, and current treatments have limited effectiveness and high costs [80]. Between 44 and 76 million of 217 million EU employees suffer from an allergic disease of the airways or skin in the European Union (EU). Up to 90% of these people are untreated or undertreated [81]. With topical corticosteroids as the primary treatment, ACD is currently a pathology without a permanent cure; the only way to improve the ailment is to eliminate the allergen as much as possible [82]. Psoriasis affects an estimated 60 million people worldwide, with country-specific prevalence being more common in high-income areas and among the elderly [35]. Psoriasis, with annual costs of over USD 11.5 billion in 2008 alone in the United States, might impose a significant economic burden on taxpayers, patients, and society in general due to its high prevalence and significant direct and indirect costs [83]. Psoriasis remains a challenge for medical systems worldwide, with expensive treatments that have short-term effects or a high number of side effects [84], and these are only a few examples of skin conditions that are a challenge for medical systems worldwide. The skin, as a therapeutic target for pCBs, represents a vast research area that has not received enough attention.

#### 2.1.1. The Skin Endocannabinoid System

The skin is the largest organ of the human body, extending over an area of 1.5 to 2 m^2^ in adults [85]. After the liver, the skin may be considered the largest organ of the human body that metabolizes drugs and xenobiotics [86]. The skin has a plethora of functions, such as body protection via the cutaneous barrier function, vitamin D synthesis, thermoregulation, and immune defense. Skin disorders affect these physiological mechanisms, oftentimes with systemic effects. It was demonstrated that skin models are valuable tools for organ-specific safety assessment concerning xenobiotic metabolism [87]. Hence, many synthetic or/and plants-based natural products may be applied to the skin for either local or systemic effects [85] with the advantage of avoiding first-pass metabolism and improving bioavailability [7]. Thus, topically applied formulations may target different skin compartments, such as the viable epidermis, known as the key target region for the majority of topical products [85]; dermis; hypodermis; or skin appendages, such as pilosebaceous units, sweat glands, and nails, while transdermal drug delivery (TDD) targets systemic circulation [85]. Hence, drug delivery into and across the skin remains challenging [88].

The skin functions as an interactive network, consisting of physical/mechanical, chemical, microbiological, and immune barriers [89]. The main components of the mechanical barrier in the skin are the *stratum corneum*, the tight junctions in the interfollicular epidermis, and pilosebaceous units [88]. Pilosebaceous units are important routes for both localized and systemic drug delivery, especially for topical liposomes [90] that are known to be appropriate as drug delivery systems even for phytocannabinoids [7]. Moreover, endocannabinoids are known to exert an important regulatory role in the biology of skin appendages [31]. CBD, a skin-permeable phytocannabinoid that has become popular in therapeutic skin products, has been discovered to have a permeability that depends on the vehicle solutions and pH of the environment, which affects its skin permeation rate and skin retention. This knowledge was obtained from in vitro research employing assays that are based on artificial membranes. [91]. Thus, CBD may have a suitable skin permeability for the development of dermatological or cosmetic applications [91]. CBD is known as a highly effective sebostatic agent that exerts its anti-inflammatory effect on human skin sebocytes [92] and on human cultured sebocytes [30]. CBD, CBN, and THC were bioaccumulated and detected using liquid chromatography–tandem mass spectrometry (LC-MS/MS) in a keratinized matrix of hair and nail samples obtained by noninvasive collection from *Cannabis* users, with significantly higher concentrations in fingernails than in toenails and hair [93]. CBD is also known to emphasize antioxidant potential by increasing the expression of the main endogenous antioxidant system, superoxide dismutase (SOD), and glutathione peroxidase (GPx) [27]. Thus, CBD is one of the most effective protectants against UVA radiation [26], protecting keratinocytes against the effects of UVA/UVB radiation by reducing lipid peroxidation products and counteracting oxidative stress [46]. CBD is also involved in wound healing [32]. Two endocannabinoids, anandamide and 2-arachidonoylglycerol, suppressed proliferation, induced apoptosis, deregulated the expression of cytoskeletal proteins (i.e., cytokeratins), upregulated lipid synthesis, and selectively activated the mitogen-activated protein kinase (MAPK) signaling pathway in human eccrine-sweat-gland-derived immortalized NCL-SG3 model cells; the overexpression of endocannabinoids levels helped manage certain sweat-gland-derived disorders, including tumors, and this was characterized by increased proliferative rates [31]. Generalized and focal hyperhidrosis or excessive sweating affects the quality of life for almost 5% of the world’s population [94]. Excessive sweating may be improved immediately after using CBD [94] and by drop administration and the inhalation of THC, which reduced the volume of sweat [95]. At the dermal level, Gerasymchuk et al. demonstrated that several pCBs exert rejuvenation efficacy and prevent cellular senescence in human dermal fibroblasts, with applications in cosmetics [32].

**Figure 1:**
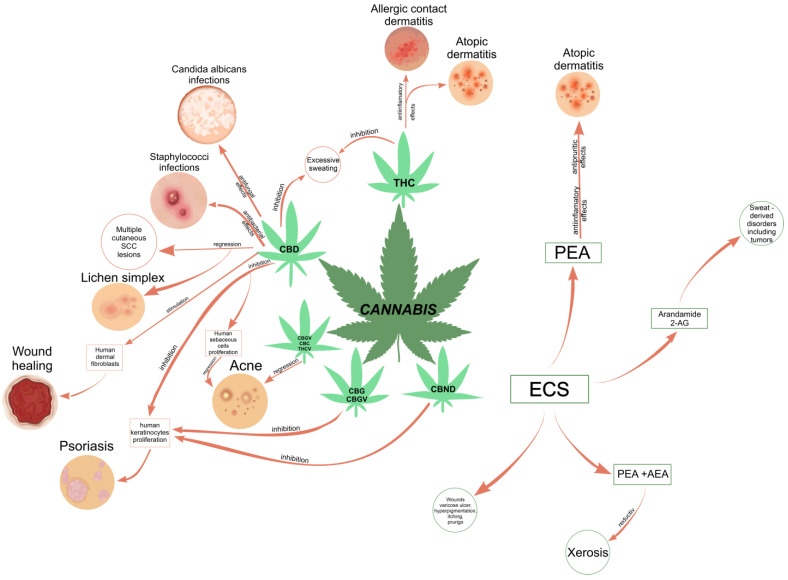
Correlation diagram between the most common phytocannabinoids and the skin conditions they could treat [30,32,51,59,93,101,106,107,108,109]. Abbreviations: THC, tetrahydrocannabinol; Δ8-tetrahydrocannabinol; Δ9-THC, Δ9-tetrahydrocannabinol; CBD, cannabidiol; CBG, cannabigerol; CBN, cannabinol; CBND, cannabinodiol; ECS, endocannabinoid system; PEA, N-palmitoyl ethanolamide; AEA, N-arachidonoylethanolamine (anandamide); SCC, squamous cell carcinoma.

The human endocannabinoid system consists of the two cannabinoid receptors CB1 and CB2; several endogenous ligands such as 2-arachidonoylglycerol (2-AG) and N-arachidonoylethanolamine (anandamide) (AEA); and enzymes involved in the synthesis, transport, and degradation of endocannabinoids [2]. Both CB1 and CB2 skin receptors, which are present in epidermal keratinocytes, cutaneous nerve fibers, dermal cells, melanocytes, eccrine sweat glands, and hair follicles [96,97,98], can be modulated by endocannabinoids, with the most studied being N-arachidonoyl ethanolamide (AEA), 2-arachidonoyl glycerol (2-AG), N-palmitoyl ethanolamide (PEA), N-alpha-linolenoyl ethanolamide (ALEA), N-linoleoyl ethanolamide (LEA), N-oleoyl ethanolamide (OEA), N-stearoyl ethanolamide (SEA), N-eicosapentaenoyl ethanolamide (EPEA), and N-docosahexaenoyl ethanolamide (DHEA) [99,100,101,102,103]. However, endocannabinoids also bind to other receptors found in various skin cells, such as transient receptor potential (TRP) channels, which are involved in a variety of processes, including the development and preservation of the skin barrier, cell growth stimulation, and cell differentiation. They also play a significant role in immunological and inflammatory processes [72]. Endocannabinoids interact with PPARs via direct or indirect signaling pathways. Biological processes like neuroprotection, anti-inflammation, and analgesic action are partially mediated by PPAR activation [59]. ECS’s involvement and role in cutaneous biology is currently an intensely debated research topic both in clinical and preclinical studies [104,105]. Topical applications of cannabinoids provide therapeutic benefits for the patient in multiple pathologies like psoriasis, eczema, and atopic dermatitis, as shown in Figure 1 [59]. These advantages are obtained due to the remarkable role of the ECS in improving dermatological conditions symptoms such as itching, inflammation, and pain.

#### 2.1.2. Skin Disorders Addressed by *Cannabis*-Based Medicines

The most common skin conditions that currently lack effective treatments that are safe and simple to administer and can significantly improve the patient’s quality of life are psoriasis, atopic dermatitis (AD), allergic contact dermatitis (ACD), asteatotic eczema (AE), acne, and seborrhea. These diseases and current treatments are detailed in Table 1. Psoriasis is a chronic autoimmune inflammatory skin disease characterized by skin lesions that are the result of a rapid turnover of epidermal keratinocyte proliferation, accompanied by the infiltration and increased expression of proinflammatory mediators in the skin [7,110]. Psoriasis incidence has a bimodal pattern, with one peak in childhood and a second peak in adulthood. It tends to persist throughout life, fluctuating in extent and severity [110]. The pathogenesis of psoriasis is complex and involves an association between genetic and environmental factors (trauma, infections, psychological stress, and drugs) [110]. The condition develops due to pathological interactions between skin immune cells and epidermal keratinocytes, leading to increased inflammation (due to the production of cytokines such as IL-17, IL-22, and TNF-α) and the excessive proliferation of keratinocytes, causing the characteristic alteration of skin called psoriatic plaque [7,57,105,110]. Cutaneous ECS inhibits cell growth and angiogenesis, leading to skin cell apoptosis, so CNBs have shown promising results in helping to treat psoriasis [7]. Clinically, psoriasis is characterized by erythematous, well-defined, raised plaques that are covered by pluristratified pearly white scales that are easily removable. The lesions are symmetrical and can affect any region, but they are more common in the extension areas—elbows, knees, lumbosacral, and pretibial regions. Psoriasis can also affect other major folds of the body and the genitals. Symptomatically, itching is mostly mild but can be severe in some patients, leading to excoriations, eczematization, and lichenification. Painful fissures may occur. After the remission of psoriasis plaques, post-inflammatory hypopigmentation or hyperpigmentation can be observed, and it is expected that these will fade in a few months [7,57,110].

In psoriasis, cannabinoids may help reduce keratinocyte proliferation. Wilkinson et al. hypothesized that the primary mechanism of the THC-mediated inhibition of keratinocyte proliferation is proliferative peroxisome-activated receptor gamma (PPARγ) [106]. A secondary inhibitory mechanism occurs via the downregulation of keratin K6 and K16 expression driven by CB1 activation [107]. Cannabinoids can prevent the release of inflammatory cytokines present in the pathogenesis of psoriasis [7]. Moreover, a synthetic cannabinoid, JWH-133, inhibits some inflammatory cytokines and angiogenic factors involved in psoriasis (inducible factor-1α (HIF-1α), vascular endothelial growth factor (VEGF), basic fibroblast growth factor (bFGF), and angiopoietin-2) in vivo and in vitro [111]. The complex action of cannabinoids is thus of great potential in psoriasis, which is justified further by their lack of the typical side effects of steroids and calcineurin inhibitors and is the most common current topical approach in this pathology.

**Table 1:** The most common skin conditions for which ECS modulation could be an effective therapeutic option.

Affection (Estimative Costs around the Current Course of Therapy)	Current Drug Therapies		Drawbacks/ Most Significant Adverse Effects	Reference
Psoriasis (between USD 23.9 and USD 35.4 billion annually in the US	topical agents	vitamin D analogues corticosteroids	poor therapy responses	[35,112]
	phototherapy	NB-UVB PUVA	major risks of skin cancer	
	standard systemic	acitretin	dry skin, hair loss, hyperlipidemia, hepatotoxicity	
		ciclosporin	hypertension, irreversible renal toxicity	
		methotrexate	bone marrow suppression, liver fibrosis, teratogenicity, hepatitis	
	biologic agents	TNF IL-17 L-23 inhibitors	individualized therapy regimen	
	small molecule inhibitors	apremilast dimethyl fumarate	used only in studies	
Atopic dermatitis (USD 3.8 billion annually in the US)	topical moisturizers	glycerin, alpha hydroxy acids, hyaluronic acid, sorbitol, urea, lanolin, mineral oil, olive oil, silicone, collagen, elastin, glyceryl stearate, shea acid, stearic acid	a large number of studies lacking any significant relevance regarding the efficacy	[113,114]
	topical corticosteroids	clobetasol, fluocinonide, betamethasone, triamcinolone, fluticasone budesonide, hydrocortisone	atrophy, striae, rosacea, perioral dermatitis, acne, purpura, hypertrichosis, alteration of the skin’s pigment, sluggish wound healing, worsening of skin infections	[115]
	topical calcineurin inhibitors	tacrolimus pimecrolimus	skin burning, increased bone growth and decreased bone density, inhibition of the hypothalamic-pituitary-adrenal axis in children	[116,117,118]
	phototherapy	UVB	xerosis, erythema, actinic keratosis, skin damage tenderness, possible carcinogenic risk	[118,119]
	systemic immunotherapy	cyclosporine azathioprine	headache, serum lipid TSH elevation, teratogenicity, liver dysfunction, leukopenia, gastric ulcer, osteoporosis, glaucoma	[120]
Allergic contact dermatitis (USD 5.3 billion in the US in 2015)	systemic corticosteroids	clobetasol, betamethasone, diflorasone, fluocinonide, halcinonide, mometasone furoate, fluocinolone acetonide, desoximetasone, triamcinolone acetonide, alclometasone dipropionate, hydrocortisone, dexamethasone, prednisolone, methylprednisolone	atrophy, infections, hypertrichosis, allergic reactions, problems with systemic absorption	[36,37,121]
	calcineurin antagonists	cyclosporine	risk of malignity	[36,38]
	ultraviolet therapy	PUVA UVB	lentigines (freckling), photoaging, precancerous lesions, actinic keratoses, skin cancer, hyperpigmentation, redness, dryness, pruritus, herpes simplex virus reactivation, polymorphic light eruption	[36,39]
Asteatotic eczema (USD 5.3 billion annually)	topical steroid	hydrocortisone, glucocorticoid, nonfluorinated steroids (hydrocortisone valerate, hydrocortisone probutate, mometasone furoate), fluorinated steroids (dexamethasone, triamcinolone, fluocortolone, flumethasone, betamethasone)	skin atrophy, striae atrophicans, xeroderma, delayed wound repair, capillary telangiectasia, rosacea-like dermatitis, steroid purpura, steroid acne milium, pigmentation disorder, acne uncontrollable, hyper-pilosis	[36,40,41]
	phototherapy	UVA (UVA-1) UVB PUVA	suppressing immune system	[40]
Acne (acne is thought to cost the economy USD 3 billion annually)	topical retinoids	adapalene, isotretinoin, motretinide, retinoyl-β-glucuronide, tazarotene, tretinoin	used in various combinations, all of these topical treatments disrupt the skin’s natural barrier, requiring frequent treatment changes	[42,43,44]
	topical antibiotics	clindamycin, erythromycin	disruption of the skin’s natural barrier	
	diverse topical agents	azelaic acid, benzoyl peroxide, chemical peels, corticosteroids, dapsone, hydrogen peroxide, niacinamide, salicylic acid, sodium sulfacetamide, sulfur triclosan	specific adverse events	
	systemic retinoids	isotretinoin	hematological/lymphatic disorders, immune system disorders, metabolic and nutritional disorders	
	systemic antibiotics	azithromycin, clindamycin, co-trimoxazole, doxycycline, erythromycin, levofloxacin, minocycline, roxithromycin	specific adverse events	
	other systemic agents	hormones, clofazimine, corticosteroids, ibuprofen, zinc sulfate	specific adverse events	
Seborrhea (in 2021, the value of the global seborrheic dermatitis market was estimated to be USD 2.2 billion)	antifungals	itraconazole, terbinafine, fluconazole, ketoconazole, pramiconazole	itching, burning sensation, dryness	[108,109,122]
	corticosteroids	prednisone	skin atrophy, telangiectasias, folliculitis, hypertrichosis, hypopigmentation	[109]
	calcineurin inhibitors	pimecrolimus	skin malignancy lymphoma	[123]
	phototherapy	UVB PUVA red and blue LED light	burning itching sensation genital tumor	[123]

Abbreviations: NB-UVB, narrowband ultraviolet B radiation; PUVA, psoralen ultraviolet A radiation; TNF, tumor necrosis factor; IL, interleukin; AD, atopic dermatitis; ACD, allergic contact dermatitis; UVB, ultraviolet B radiation.

Atopic dermatitis (AD) is a chronic, relapsing, pruritic condition that is often associated with allergic rhinitis and/or asthma. Infants and children are most often affected, with 85% of cases appearing in the first year of life and 95% of cases appearing by 5 years [110]. The pathogenesis of AD is not fully understood, but it is believed that genetic and environmental factors influencing epidermal barrier function and adaptive immune function are involved [7,57,110]. Genetic mutations of various genes/proteins are investigated, such as filaggrin mutations, which are present in 50% of cases, causing a defective skin barrier and increased transepidermal water loss–dry skin. Deficiencies in ceramides, antimicrobial peptides, and altered sensation to itch stimuli are also documented. AD involves a type 2 cytokine inflammatory response with the activation of IL-4 and IL-13 [57,110]. Actual treatments for AD are topical moisturizers, corticosteroids, calcineurin inhibitor, phototherapy, and systemic immunotherapy [113,114,115,116,117,118,120]. All of these treatments have limited short-term efficacy, significant or even limiting side effects when administered for long periods of time, or are prohibitively expensive [113,114,115,116,117,118,120].

AD can be divided into three stages: infantile AD, occurring from 2 months to 2 years of age; childhood AD, occurring from 2 to 10 years; and adolescent/adult AD. In all stages, pruritus (itching) is a hallmark of atopic dermatitis, often preceding the appearance of lesions. Clinical features include intense itching, scratching and eczematous lesions that can be acute (erythema, vesicles, bullae, weeping, and crusting), subacute (scaly plaques, papules, round erosions, and crusts) or chronic (lichenification, scaling, and hyper- and hypopigmentation). Acute inflammation with the involvement of the cheeks, scalp, and extensor areas of the extremities predominates in infants, and chronic inflammation with lichenification is preferentially located in flexural areas in children and adults. Follicular patterns of atopic dermatitis (follicular eczema) are more common in persons with darker skin phototypes. The condition has a severe impact on the quality of life due to disturbed sleep, poor performance at school or work, and unsightliness [110].

Despite the complexity of the underlying mechanisms, there are intriguing reports that CB receptor agonists have improved AD symptoms. Cannabinoids have demonstrated anti-inflammatory and antipruritic properties, highlighting a potential therapeutic role [7]. The mechanisms by which cannabinoids decrease inflammation and pruritus are diverse and involve CB1/CB2 receptors, chemokines, and an interaction between the endocannabinoid system and the immune system [7,124]. Nam et al. showed that a CB1 agonist ameliorates AD in mouse models possibly by decreasing mast cell proliferation [121]. Clinical trials have also shown promising results for AD [122]. In a study with pediatric and adult subjects, a cream containing palmitoylethanolamide (PEA) significantly increased the mean time to the next eruption [123]. Similarly, in a pilot study, the use of a topical cannabinoid emulsion resulted in clinical resolution and prevented the relapse of mild atopic dermatitis in 80% of patients [124]. In another study, a cream containing PEA improved itching severity and sleep quality by an average of 60% among subjects [125]. With both anti-pruritic and anti-inflammatory effects, this PEA product is an alternative to traditional steroids, anti-histamines, and calcineurin inhibitors, and it has a superior safety profile in addition to its good efficacy.

Allergic contact dermatitis (ACD) is a pruritic, eczematous reaction and one of the leading causes of occupational diseases [110]. ACD represents a delayed-type (type IV) hypersensitivity reaction that occurs when allergens activate antigen-specific T cells in a sensitized individual. ACD typically requires repeat exposures before an allergic response is noted and can occur 24–48 h after exposure to the offending agent. This exposure induces a specific immune response, predominantly involving T cells and inflammatory cytokines such as interleukin IL-6 and IL-8 and tumor necrosis factor-alpha (TNF-α). There is also an endogenous predisposition that relates to the malfunctioning of epidermal proteins such as filaggrin, ceramides, claudins, antimicrobial factors, and proteases [110]. Clinical patterns will vary based on the actual allergen involved, but the typical appearance is often a well-demarcated pruritic eczematous eruption, which may be acute (blistering, weeping, and/or edema) or chronic (lichenified or scaly plaques). This reaction is typically localized to the area of skin that comes into contact with the allergen. ACD may coexist with irritant contact dermatitis, contact urticaria, and various forms of endogenous eczema, which can complicate establishing an accurate diagnosis [7,110,125]. There are numerous potential contact allergens, but the most common are metals (e.g., nickel, cobalt, and chromates), fragrance, preservatives (e.g., methylisothiazolinone), formaldehyde and formaldehyde releasers (e.g., quaternium-15), paint p-phenylenediamine, topical antibiotics (e.g., neomycin and bacitracin), and rubber accelerators [110].

Treatment for ACD consists of avoiding trigger factors and using topical corticosteroids, calcineurin inhibitors (tacrolimus and pimecrolimus), and systemic immunosuppressive agents for severe cases [110]. Regarding the therapeutic value of using CNBs in the treatment of ACD, human studies are still lacking, but studies in mice have revealed the involvement of CBRs, especially CB2R, in the inflammatory response of ACD, and possible therapies involving it have been proposed targets [57,126].

As with AD, studies have shown that phytocannabinoids are effective in the treatment of ACD. In a rodent study, the topical application of THC reduced inflammation in ACD by inhibiting keratinocyte-derived proinflammatory mediators (CCL8 and CXCL10) independent of CB1/CB2 receptor activation or inhibition [124]. Kim et al. designed a structurally similar compound to anandamides, which improved epidermal barrier function and suppressed TH2 cytokine expression in mice, also activating CB1 receptors [127]. CBD showed anti-inflammatory properties in an ACD experimental model in a different study by Petrosino et al. [128].

Asteatotic eczema (AE), also known as craquelé eczema, winter itch, or xerotic eczema, is a common type of pruritic dermatitis characterized by dry, scaly, cracked, and itchy skin [110]. In terms of pathogenesis, the xerosis of aged skin is not caused by deficient sebum production but by a complex dysfunction of the stratum corneum. A decrease in intercellular lipids is present with a deficiency of all key lipids in the stratum corneum and an altered ratio of esterified fatty acids to ceramide, plus the persistence of corneodesmosomes and the premature expression of involucrin with cornified coat formation, resulting in marked corneocyte retention [110,126]. As a result, the stratum corneum dehydrates, loses its flexibility, and forms small cracks, which make the surface of the skin dull, rough, and scaly. Mild xerosis is asymptomatic, but if it is more pronounced, the skin transmits unpleasant sensations such as itching and stinging. Inflammation is intensified by the release of pro-inflammatory cytokines that are secondary to barrier disruption, mechanical factors (scratching and friction), and the application of irritants or sensitizers in topical preparations or skin care products. Prevention is key in avoiding or controlling itching and irritation, so patients are advised to make several lifestyle changes, such as avoiding harsh cleansers and opting for lukewarm showers, to prevent exacerbation of this uncomfortable disease [57,110,126]. Clinical xerosis usually has a slow and indolent course that progresses over the years. It is characterized by dry, dull, and rough skin with fine bran-like scales that flake off easily. In contrast, eczema craquelé usually has a more acute or subacute onset. It is characterized by redness and tight-appearing polygonal cracked skin with fine, interconnected horizontal and vertical fissures. This forms an irregular network of fissures and cracks, similarly to broken window glass. It is most severe on the distal legs and occasionally the arms and trunk. The face, scalp, groin, and axillae are usually spared from the fine, dry scales of the condition. Crusting, oozing, and bleeding fissures may be observed in advanced cases [57,110,126].

Treatment for AE usually involves the application of emollients containing urea and lactic acid, and severe cases usually require topical corticosteroid treatment [110]. Endocannabinoids such as PEA and AEA exist in high concentrations in the granulosa layer of the skin, and low levels of these compounds have been linked to xerosis. Several studies have provided evidence that the modulation of the cutaneous ECS can lead to increased lipid synthesis in that skin layer, providing relief in eczematous conditions [126,129].

Acne and seborrhea are the most common dermatological conditions, and they are usually self-limiting and characterized by a very high production of sebum (lipids) and chronic inflammation of the pilosebaceous unit. It is especially common in teenagers, but it also occurs in children and adults, and its psychological impact can be profound [110]. The pathogenesis involves multiple factors, including (i) increased sebum production, (ii) follicular hyper-keratinization and corneocyte hyper-cohesiveness, (iii) the proliferation of the bacterium *Cutibacterium acnes* (formerly known as *Propionibacterium acnes*), and (iv) inflammation that is neutrophil driven in early lesions and Th1/Th17 driven in established lesions. Acne and seborrhea typically begin at puberty as a result of the androgen stimulation of the pilosebaceous unit and changes during keratinization at the follicular orifice [57,110,126]. Acne is characterized by a wide spectrum of lesions, such as open and closed comedones (blackheads and whiteheads); inflamed papules and pustules; nodules and pseudocysts in severe acne; and post-inflammatory erythematous or pigmented macules. Acne can be classified as mild, moderate, or severe, but this designation may vary between clinicians as there is no single grading system that has been adopted by all. Acne vulgaris is most commonly found on the areas of skin with the greatest density of sebaceous follicles, such as the face, back, and upper chest. While in a benign condition, acne can lead to permanent scarring and significant psychosocial distress. Therefore, the initiation of treatment in the earliest stages is preferable [110]. Acne treatments are aimed at targeting as many pathogenic factors as possible to control the disease. Depending on the stage of acne, the therapeutic management involves the use of the following: (i) mild acne: benzoyl peroxide, topical retinoid (tretinoin, tazarotene, adapalene, and trifarotene), and topical combination therapy (benzoyl peroxide plus a topical antibiotic, a topical retinoid, or both); (ii) moderate acne: topical combination therapy, oral antibiotic (doxycycline, minocycline, and tetracycline) plus topical retinoid and oral antibiotic plus topical retinoid plus benzoyl peroxide; (iii) severe acne: oral antibiotic plus topical combination therapy, oral isotretinoin, and hormonal therapies (female patients)– spironolactone and oral contraceptives. Cannabidiol has been suggested as a promising therapeutic agent for the treatment of acne vulgaris because it normalizes the lipogenesis of sebocyte cells, decreases the proliferation of these cells, and decreases the levels of pro-inflammatory cytokines [105,110,129].

Endocannabinoids have shown promising results for the treatment of acne when CB2 expression was found in human SZ95 sebocytes [130]. The inhibition of CB2 receptors resulted in the suppression of basal lipid production, suggesting that CB2 antagonists may be helpful in the treatment of skin conditions characterized by the dysfunction of the sebaceous gland [100]. In a single-blind study involving 11 patients, Ali et al. investigated the use of a cream containing 3% cannabis seed extracts for acne. Participants used the cream twice daily for 12 weeks, and their sebum production significantly improved [131]. Cannabidiol inhibits sebocyte proliferation by activating TRPV4 according to Olah et al. In cultured human sebocytes, it also inhibits the lipogenic effects of arachidonic acid, linoleic acid, and testosterone [101]. Thus, with future studies to be conducted, cannabinoids could be included in the daily management of this pathology, where the currently available options are far from optimal efficacy and safety.

Wounds, varicose ulcers, hair loss, hyperpigmentation, itching, prurigo, and lichen simplex are a few other skin conditions that have been linked to disruptions of ECS, as per recent studies [97,98,106]. The range of dermatological conditions in which ECS becomes a therapeutic target is widened by the existence of specific cannabinoid receptors at hair follicles and sweat glands level [59]. The mechanisms by which cannabinoids ameliorate inflammation and pruritus are various and involve, in addition to CB1/CB2 receptors, chemokines and interactions between the endocannabinoid system and the immune system [132].

It has been shown in several studies that by inhibiting TRPV1, anandamide had antipruritic effects [133,134]. The cutaneous itch-inducing TRPV1 ion channel is primarily found in the nociceptive neurons of the peripheral nervous system. Twenty-two patients with pruritus, prurigo, and lichen simplex were treated by Stander et al. using PEA incorporated into an emollient lotion. PEA, which inhibits fatty acid amide hydrolase enzyme (FAAH) and activates anandamide at the cannabinoid receptor, reduced itching in these patients by 86.4% [135]. The treatment of uremic pruritus with cannabinoids has also been shown to be successful. In a 3-week study, Szepietowski et al. investigated the efficacy of a cream containing lipids and endogenous cannabinoids in 21 patients with uremic pruritus. According to their findings, 81% of patients had completely reduced xerosis, and 38% of patients had completely resolved itching [136].

Wound healing is a difficult process that involves three overlapping phases: inflammation, proliferation, and tissue remodeling [137,138]. Endocannabinoid system signaling may affect the intricate process of wound healing because it regulates fibroblast function, epithelial differentiation and proliferation, and skin inflammation. The involvement of CB1 and CB2 receptors during the wound-healing process in various immune cells and fibroblasts was demonstrated using murine models [139,140,141]. Different cannabinoid analogs produced a wound-healing response in these models, which may have been mediated by the activation of CB1 and/or CB2 receptors, increasing anti-inflammatory factor synthesis, the indirect activation of TRPV1 and epidermal growth factor receptors, and the inhibition of the FAAH enzyme [140,142]. There is not much research supporting the use of phytocannabinoids, specifically CBD, for the treatment of wounds. Three patients with epidermolysis bullosa, a rare skin condition marked by pain and blisters, had faster wound healing, fewer blisters, and pain relief with respect to self-reported topical cannabidiol use [60,143]. Although there is a lack of clinical evidence, preclinical models indicate a promising future not only due to their efficacy but also based on its excellent safety profile compared to many of the classical options.

The effects of CBD and *Cannabis sativa* extract (CSE, standardized to 5% CBD) on human keratinocytes (HaCaT cells) and human dermal fibroblast (HDF) cells were examined in a study by Sangiovanni et al. [144]. The TNF-α treatment of human keratinocytes increased the expression of 26 genes involved in inflammatory pathways, including chemokines like CXCL8 and CXCL10; interleukins like IL-17C and IL-1B; and VEGF-A. CSE treatment reduced all 26 genes related to inflammation, while CBD alone reduced only 15 genes. TNF-α treatment increased the expression of 16 genes that are essential for wound healing in HDF cells. CBD had no inhibitory effect on genes that are involved in inflammation and matrix remodeling, such as IL-6 and MMP-9, while CSE was once again able to reduce all genes. These findings suggest that the other cannabinoids, flavonoids, and terpenes present in CSE may have a synergistic anti-inflammatory effect that is greater than CBD alone [144].

Varicose ulcers are extremely common skin wounds on the lower limbs that, despite the use of compression therapies, are still challenging to heal. In a recent study, compression bandages were applied in combination with topical cannabis-based medications to the wound bed and surrounding tissues to treat nonresponsive lower limb ulcers in 14 patients with an average age of 75 years [50]. Complete wound closure, defined as fully epithelialized, was reached in 79% of patients, and 81% of wounds healed in a median time of 34 days. The remaining patients had promising healing trends but were unable to be followed up on. The rapid wound closure of nonhealing venous foreleg ulcers in elderly and highly complex patients suggests that topical cannabis-based medications may be useful therapeutic approaches when combined with compression therapy [61]. The applicability could also be extended to the management of other types of wounds.

### 2.2. The Skin as a Potential Route for Systemic Administration

**Figure 2:**
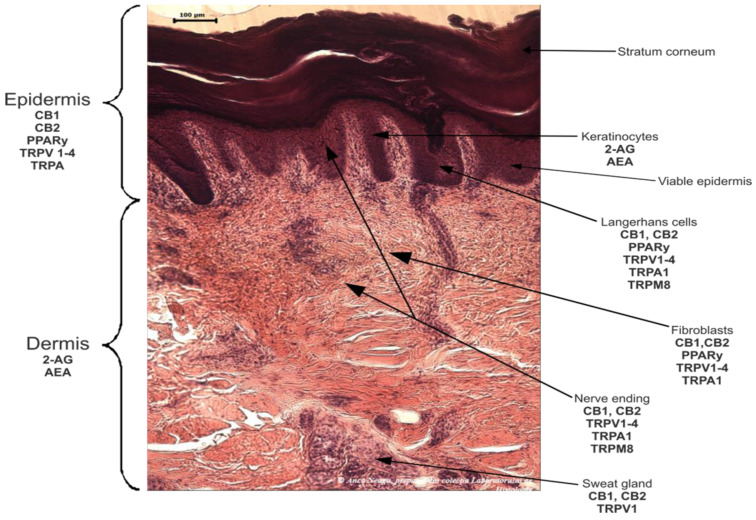
The molecular members of the skin’s endocannabinoid system (ECS).

**Figure 3:**
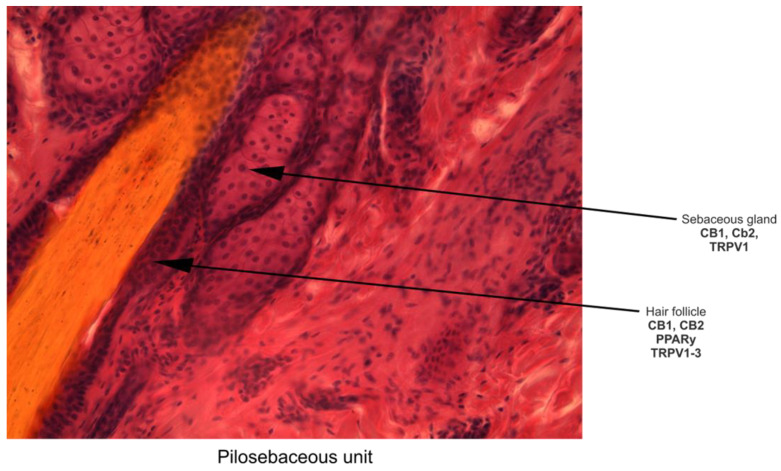
Particularities of skin ECS at the pilosebaceous unit level.

The skin, which is the largest organ in the human body and serves as a barrier between the body and the outside world as well as an immunological function, is made up of three layers, the dermis, the epidermis, and the stratum corneum (SC) [145,146,147,148], as shown in Figure 2 and Figure 3.

The skin has unique characteristics: It is permeable relative to the environment and allows the diffusion of air, heat, water, and low molecular weight molecules [145]. Skin permeability can occur in three different ways: the intercellular pathway, which involves the space between corneocytes and keratinocytes; the transcellular pathway, which involves corneocytes and the lipid matrix around them; and the appendicular pathway, which involves sweat glands and hair follicles [149]. Because of this, transdermal application can be used as an alternative method of drug administration. This is because it is simple to use and because it is administered directly into the systemic circulation, avoiding the first hepatic passage and the potential appearance of unwanted metabolites; moreover, the necessary dose and side effects can be significantly reduced [73,150,151].

The transdermal administration of cannabinoids avoids the first-pass metabolism that occurs in the liver, but because they are extremely hydrophobic, they have a limited diffusion across the aqueous layers of the skin [152]. From a physicochemical perspective, molecules with a low weight (below 500 g/mol), a melting point of less than 250 °C, and high potency (less than 10 mg/day) are suitable for transdermal administration. Because transdermal administration necessitates passing through the hydrophobic/lipophilic SC, absorption in the deep aqueous layers, and then passage into the systemic circulation, these substances also need to have a moderate lipophilicity/hydrophilicity balance (logP = 1–5) [73,150,153]. Because the molecular weight of cannabinoids is not what we want, nor is the ratio of lipophilicity/hydrophilicity, they have a log P between 5 and 7 (as seen in Table 2) and low solubility in water; their transdermal administration represents a challenge. These difficulties not only led to the use of permeability enhancers such as oleic acid and ethanol but also techniques that modify the characteristics of the molecule by using microemulsions, physical amplifiers (microneedles and electroporation), iontophoresis, ultrasound, magnetophoresis, encapsulation in micro-/nanogels, nanoparticles, and nanocarriers [25,73,76].

#### 2.2.1. Why Transdermal Administration of Cannabinoids Could Be the Solution?

Modern medicine is constantly looking for therapeutic methods that bring definite improvements in the clinical condition of patients. At the same time, researchers are looking for new drugs that have a high degree of safety when administered and, last but not least, can cover a wide range of ailments.

Therefore, cannabinoids are a class of compounds that seem to satisfy these needs via the multiple proven or still researched therapeutic effects, such as the following: analgesic, anti-inflammatory, antioxidant, antibacterial, and antiemetic [45,62,159,160,161]. Cannabinoids have sparked the interest of scientists due to their high therapeutic potential. For this reason, pharmacological studies address subjects that debate and analyze the methods of the administration of cannabinoids to improve their bioavailability, ease of administration, and reduction in adverse reactions. For example, the most intensively analyzed route of administration is the oral one. Additionally, recent studies have highlighted that cannabinoids have low bioavailability following oral administration. Some of the reported causes are as follows: (1) the high lipophilicity of these compounds such that low solubility in water that limits their absorption, (2) hepatic first-pass metabolism, (3) rapid metabolism, (4) irregular pharmacokinetics, (5) their instability in the presence of gastric pH, and (6) the influence of temperature [76,162,163,164]. Considering the above-mentioned reasons, it is expected that researchers will explore other routes of administration in an attempt to minimize these inconveniences. Therefore, transdermal formulations come to the aid of scientists. In other words, theoretically, problems arising from the oral administration of cannabinoids can be solved by using the skin as a way of entering the body when the pathology or symptom being treated allows it. Some disadvantages of cannabinoid oral administration are detailed in the following lines.

The effect of the first hepatic passage is manifested by the biotransformation of the drug before it enters systemic circulation. Both endogenous and exogenous cannabinoids undergo hepatic metabolism after oral administration, a fact that considerably influences the bioavailability of the drug. Specifically, cannabinoids are substrates of the CYP enzyme family [165]. For example, dronabinol (Marinol), a synthetic delta-9-THC for oral use, is primarily metabolized by hydroxylation via multiple CYPs. Both active and inactive metabolites result from this reaction [166]. An in vivo study on patients treated with CBD identified the presence of 33 different metabolites in the patients’ urine using spectrometric methods. Thus, due to the intense metabolism undergone by CBD, the study suggests that this natural compound could provide valuable information about the mechanism of several types of biotransformation [167].

What is interesting is the fact that the metabolism of cannabidiol occurs primarily due to hydroxylation, which is catalyzed by CYP2C19 and CYP3A4 enzymes [168]. In addition, CBD is a potent inhibitor of these enzymes [169]. This information is significant in the context of the simultaneous administration of the Epidiolex product (synthetic cannabidiol: GW Pharmaceuticals) as an adjuvant in patients with refractory epilepsy treated with clobazam, which is another substrate for these enzymes that belong to the CYP family. Consequently, the plasma level of the active metabolite of clobazam (norclobazam) requires frequent monitoring in patients following this treatment simultaneously with CBD [169].

Regarding the metabolism of endocannabinoids, studies have shown the existence of some inactive metabolites. In addition, research has focused on inhibiting the degradation of endocannabinoids with pharmacologically valuable results. Therefore, it is interesting to mention FAAH inhibition strategies, with this molecular target becoming extremely attractive for the anxiolytic effects observed in neuropsychiatric diseases and the antinociceptive effect in pain conditions [170]. Thus, FAAH inhibition has been intensively explored in clinical and preclinical studies [171]. For example, modulating the concentration of anandamide by inhibiting FAAH can reduce anxious behavior in rodents with chronic stress [172]. In addition, in a multicenter, double-blind, placebo-controlled study, JNJ-42165279, an FAAH inhibitor, reduced the Liebowitz Social Anxiety Scale (LSAS) by 30% and achieved an improvement in the Clinical Global Impression-Improvement (CGI-I) by the end of 12 weeks of treatment [173]. Therefore, it is easy to understand that the hepatic metabolism of orally administered cannabinoids produces qualitative and quantitative changes in the active substances. Also, the substrate–enzyme interaction increases the risk of drug interactions, to which the risk of toxicity or failed treatment is added.

Also, cannabinoids are molecules with a high degree of lipophilicity and are susceptible to degradation if they are formulated as solutions. They can undergo autoxidation reactions; thus, control over the pharmacokinetics of the administered compound is lost. In addition, exposure to light, herbal cannabis or cannabis resin, or extracts leads to significant losses in phytochemical component levels [174,175].

Their high lipophilic nature poses serious problems in cannabinoid oral administration. It is well documented that the oral bioavailability of CBD and THC is as low as 6% [175]. Also, THC under various oral formulations shows variable absorption. Moreover, THC absorption is slow, irregular, and unpredictable. Pharmacokinetic studies have noted similar oral absorption for both THC and CBD, a fact that requires the formulation of these compounds in oil to counteract the harmful effects on oral bioavailability given the low solubility of cannabinoids in water [25].

On the other hand, the variable pharmacokinetics of cannabinoids is demonstrated by a significantly different metabolic pattern depending on the oral formulation [176]. For example, in a double-blind, randomized, single-center study, nine healthy male volunteers were treated with unheated *Cannabis* sp. extracts, heated *Cannabis* sp. extracts, and dronabinol. The results of the study demonstrated the different plasma concentration levels of certain metabolites depending on the form of administration, in addition to the higher bioavailability of the heated extract compared to the unheated extract and synthetic CBD. Specifically, the authors found the highest scores of the average plasma value of the area under the plasma concentration/time curve (AUC) during the first 24 h after drug administration for THC-COOH, which is the inactive metabolite of dronabinol [177].

Consequently, the results of the oral administration of cannabinoids impose pharmacokinetic problems that are difficult to anticipate and require rigorous comparative studies on the medicinal form approached. Also, the variable bioavailability of cannabinoids requires the development of quality-controlled extracts, with a pharmacological profile that allows the administration of correct doses for optimal therapeutic effects.

That is why the transdermal administration of cannabinoids would have some advantages: the possibility to increase the therapeutic dose by avoiding the hepatic first-pass effect; direct application to the affected area, especially in dermatological conditions or the possibility to obtain an analgesic effect; and adverse effect minimization, as seen in Table 3. On the other hand, transdermal preparations allow the use of larger amounts of active substance, with controlled release for a longer period of time [178]. However, there are several difficulties and restrictions with respect to transdermal cannabinoid preparations. The physico-chemical stability of the preparation is a subject that deserves attention in this context.

#### 2.2.2. The Use of Nanotechnologies for Cannabinoid Transdermal Administration

**Figure 4:**
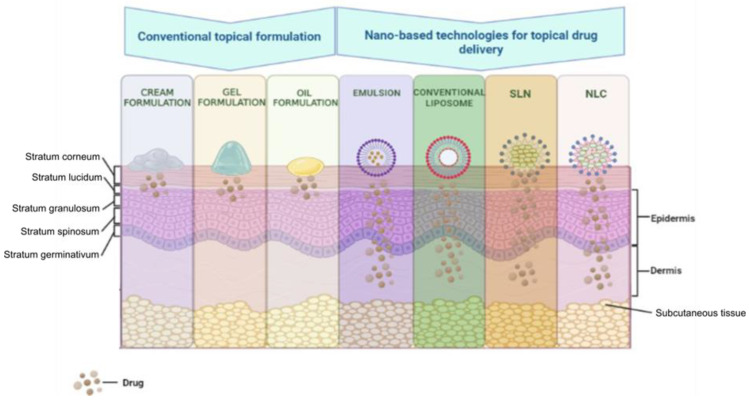
The passage of substances through the layers of the skin depending on the way they are formulated. SLN, solid lipid nanoparticles; NLC, nanostructured lipid carriers.

The field of cannabinoid research is expanding its boundaries more and more. The understanding of the pharmacology of these compounds and the knowledge of their beneficial effects in the treatment of pain, post-chemotherapy emesis, neuropsychiatric conditions, and dermatological conditions, in particular, stimulated researchers to address topics related to optimal formulations. Theoretically, the most promising formulations, which exceed the limitations imposed by the physico-chemical characteristics of cannabinoids, mentioned above are those that allow their administration on the skin [154]. Among these skin administration methods, we focus on those based on nanotechnology, that have, as observed in Figure 4, better penetration through skin layers than conventional formulations.

These formulations include but are not limited to lipid-based nanocarriers (liposomes, nanostructured lipid carriers, and solid lipid nanoparticles) and vesicular nanocarriers (niosomes, liposomes, transferosomes, ethosomes, and transethosomes) [210].

Due to the high lipophilic nature of cannabinoids, creams are one of their topical administration systems with multiple advantages. The topical application of cream containing CBD and CBD:THC in various proportions allowed a reduction in the symptoms of epidermolysis bullosa simplex and psoriasis [211,212].

However, creams incorporating cannabinoids have their limitations. To increase the stability of active substances, it is necessary to ensure a slightly acidic pH. CBD, for example, degrades at pH = 6 and pH = 7. In addition, increasing the stability of the cannabinoid using alcoholic formulations, as required by the physico-chemical characteristics of these compounds, is not recommended for applications to the skin, especially in dermatological conditions [174,213]. In addition, light and temperature must be controlled so that the product can keep the properties for which it was created [91].

Another delivery system for cannabinoids through the skin, with a series of improved characteristics compared to oils and creams, is to use gels. From a mechanistic point of view, gels allow the deep penetration of cannabinoids through the skin for a systemic effect. It is interesting that researchers have tried to apply different techniques to obtain constant and controllable concentrations of cannabinoids in the plasma. In this sense, in a study on a rat model of arthritis, the gel containing CBD reduced the associated inflammation, limb posture score, and thickening of the synovial membrane in a dose-dependent manner [214].

Advanced research has allowed the improvement of these forms of administration so that both gels and creams have been proposed to improve their characteristics using nanotechnology. Thus, improving the contact time with the skin, producing a uniform dispersion of the active substance, increasing the stability and bioavailability of the active substance, and high penetration through the skin are some of the qualities of the formulations improved by nanotechnology [215].

Nanoemulsion is “a system of water, oil, and amphiphile, which is a single optically isotropic and thermodynamically stable liquid solution” [216]. This type of emulsion is a formulation option that is suitable for both lipophilic and hydrophilic substances [217]. The advantages of nanoemulsions include reduced toxicity, the ability to be administered by various routes (topical, transdermal, parenteral, and others), and an increase in the stability of incorporated active substances [215,217]. Using this release system, numerous pharmaceuticals, including analgesics, anti-inflammatories, antidepressants, anti-anginal, anti-psychotics, and anti-cancer agents, have been researched and developed [217]. Recently, nanoemulsions significantly improved the skin penetration capacity of THCA and CBDA cannabinoids. This transdermal release system potentiated the stability, permeation rates, and solubility of cannabinoids. Thus, these promising results propose nanoemulsions as a strategy to improve the stability of cannabinoids in dermal formulations [189]. On the other hand, although promising, this drug delivery system also presents a series of disadvantages. The realization of nanoemulsions requires a series of special formulation techniques, which involve considerable financial efforts. In addition, there is scientific evidence that confirms a potential disruption of the integrity of the lipids of the corneous layer of the skin, a disadvantage generated by substances that further enhance the permeation of the active substance through the skin [215]. Despite these drawbacks, the research field of nanocarriers continues to develop. Thus, other nanoparticles are created, such as liposomes, to improve drug delivery skin systems.

One or more lipid layers that incorporate an aqueous phase structurally form nanoparticles called liposomes. These vesicular nanocarriers have the advantage of allowing both the topical and transdermal administration of drugs. In addition, liposomes allow the controlled release of the active substance [215]. According to some researchers, by having a higher skin deposition than other delivery systems, liposomes have superior properties relative to oil-/water-type emulsions and solutions [218].

From a mechanistic point of view, liposomes improve the topical administration of drugs by acting as a rate-limiting barrier, which prevents systemic absorption where this is not desired. Additionally, by improving the flexibility of liposomes with the help of surfactants and adding elastic lipid bilayers, these nanocarriers can serve for the transdermal administration of drugs with promising results [219]. At the same time, this delivery system allows the incorporation of highly lipophilic substances, such as cannabinoids. A recent study on an experimental canine model of chronic pain tests the subcutaneous administration of CBD in the form of liposomes. This study demonstrates that liposomal CBD produces qualifying CBD plasma concentrations that are maintained over the tested period (28 days) [220]. Accordingly, liposomes can become a successful method of administration for cannabinoids in the future due to their proven analgesic effect. However, there are also disadvantages with respect to the use of these nanocarriers. These formulations can determine the systemic absorption of active substances through the shunt or follicular pathway. Thus, there is a risk of systemic adverse reactions. In addition, the high costs of the preparation technology used and certain stability problems can create difficulties in the clinical expansion of these types of formulations [219,221].

Solid lipid nanoparticles (SLNs) are lipid nanoparticles that are known for their low toxicity, ensuring biodegradability and biocompatibility for drug delivery. In addition, via topical administration, these systems potentiate the penetration of active substances through the SC of the skin, ensuring a uniform, homogeneous administration and increased adhesiveness. On the other hand, these systems have the disadvantage that they can lead to drug deposits in certain areas of the body where they were administered. The desired drug deposits, which have therapeutic importance, are at the level of hair follicles and sebaceous and sweat glands [215]. These nanocarriers are superior to nanoemulsions and are suitable candidates for the incorporation and topical administration of highly lipophilic drugs [222].

On the other hand, nanostructured lipid carriers (NLCs) are a form of nanoparticles with improved properties compared to SLN. These nanocarriers are made up of a mixture of solid and liquid lipids. These nanoparticles show promising results for topical administration. They form small-sized particles, have superior adhesion power at the level of the SC, increase the bioavailability of drugs, and improve the hydrophilic properties of the active substances. Compared to SLNs, NLCs have improved occlusive properties [223,224]. Unfortunately, the evidence for the effective transdermal administration of NLC is insufficient and inconclusive [225]. Cannabinoids have been studied in the form of NLC. For example, the intranasal administration of CBD in the form of NLC improved antinociceptive effects in chronic neuropathic pain induced by chemotherapy in a murine model. This effect was more potent for NLC compared to the CBD solution [207]. Additionally, in a recent in vitro study, it was shown that the photostability of cannabidiol extracts can be improved by their encapsulation in nanostructured lipid carriers (NLCs). In addition, in the same study, the authors managed, using this type of nanoformulation, to potentiate the anti-inflammatory activity of cannabidiol at this level [162].

In summary, drug delivery systems that use nanotechnologies present both pros and cons. Even so, lipid-based nanotechnologies are intelligent drug delivery systems and are promising for the administration of cannabinoids on the skin for topical and transdermal action.

## 3. Future Perspectives on the Transdermal Delivery of Cannabinoids

The undeniable development with respect to increasing cannabinoid bioavailability, whether natural or synthetic, has increased their potential of being introduced into therapy, with the ultimate objective of increasing bioavailability and efficacy and lowering toxicity and/or adverse effects. However, as the research study’s findings indicate, highlighted in Section 2 of the current work, such studies need to continue to provide feasible solutions to the issues that scientists in the field are still facing, and these issues are as follows: (i) most cannabinoids have harmful and/or psychoactive effects when administered systemically; (ii) the need for extending the pain-relieving effect; (iii) the poor ability of cannabinoids within topical formulations to cross all skin layers; (iv) overcoming the legal issues associated with the use of these substances for therapeutic purposes.

Cannabinoid study has become a mainstream discipline as a result of the growing interest in the use of cannabinoids in pharmaceutical formulations, which show promise in the treatment of a number of diseases. These studies revealed a couple of key concepts that need to be considered when designing or optimizing novel pharmaceutical formulations with the purpose of enhancing cannabinoid transport. These concepts will be further discussed in more detail. Using cannabinoid compounds with no affinity for CB1 receptors from CNS (central nervous system), which drastically limits the selection of effective solutions, or formulating them so that they cannot cross the BBB are two practical ways to avoid the psychotropic action of cannabinoid compounds. Another interesting perspective in order to avoid the psychotropic effects of common cannabinoids is represented by the design and synthesis of dualsteric/bitopic ligands that are able to selectively target CB2 receptors versus CB1, as Gado et al. reported [226].

When released into the bloodstream, cannabinoids have the ability to cross the BBB, where they will exert their undesirable effects on the CNS. The topical formulations of such substances may thus offer a unique alternative to avoid their binding to CB1 receptors at the central level by employing the structures of the epidermis as a reservoir for cannabinoids. This paradigm is obviously particularly important, as it prevents the rapid release of a large amount of substance into the bloodstream with the detrimental consequences already mentioned, but it is equally important to develop formulations that enable the controlled release of the active ingredient from these deposits as a way to extend the duration of the expected effect.

Pharmaceutical formulations for external application need to ensure effective transdermal delivery of the therapeutically relevant substance. In this regard, the oil–water partition coefficient, logP, which describes the degree of lipophilicity of a molecule or molecular assembly [227,228], is of particular interest, along with other physicochemical properties, including the melting point, aqueous solubility, pKa, and molecular weight (Mw). In order to be able to penetrate through the skin’s layers, particularly the SC, the optimal log P value of a chemical entity should be within the range of 1–3 [229,230]. Consequently, transdermal formulations of cannabinoids should reduce their log P from values of 6–7 to those within the optimum range.

As revealed in Section 2.1, the ongoing research with respect to obtaining appropriate solutions for the use of cannabinoids for therapeutic purposes has resulted in the development of a wide range of formulations, with each one having its advantages and disadvantages. The most promising outcomes from the perspective of therapeutic efficacy which don’t cause significant discomfort for the patient and at a competitive production price, should serve as a basis for the further development of technologies envisioned for introduction the of new cannabinoid therapeutic agents into the clinic. Nanotechnology allowed the encapsulation of cannabinoids in nanocarriers, improving their physical–chemical stability and bioavailability while also preventing degradation. In this regard, several nanotechnology-based formulation strategies have been developed, of which lipid-based nanoparticles have proven to be the most attractive for transdermal delivery. As shown in Figure 4, such formulations can be tailored in various approaches, and these range from simple micro- or nanoemulsions to complex architectures, such as liposomes, solid lipid nanoparticles (SLNs), lipid nanocapsules (LNCs), or self-emulsifying drug delivery systems (SEDDS) [22,231]. Despite the fact that they exhibit obvious benefits over conventional topical formulations, typical liposomes are believed to be unsuitable as transdermal delivery systems of drugs. Unmodified liposomes have been retained in the SC because of their rigid structure, which prevents diffusion into the deeper skin layers. In addition, the use of regular liposomes is limited by their poor stability, poor encapsulation efficiency, and decreased loading capacity [232]. To develop an innovative class of liposomal formulations with enhanced properties, several approaches have therefore been evolved, with one of them being focused on deformable liposomal formulations including transfersomes, ethosomes, invasomes, mentosomes, and niosomes. Ogunsola and colab. have depicted a method for the preparation and characterization of flexible liposomes, also named transfersomes, and they are able to pass through the small intercellular spaces between SC cells (20–40 nm) [233]. This study has established the dependence between the degree of skin permeability and the ratio between phosphatidylcholine (PC) and Tween 80 (a non-ionic surfactant), the main constituents in the composition of the liposomes prepared by the research group. To be included in transdermal formulations, future research should focus on optimizing the preparation method of this type of liposomes to counteract their main weaknesses, such as instability against oxidative degradation [234].

Sermsaksasithorn and colab. have approached the transdermal administration of CBD from *Cannabis sativa* with the help of a transdermal patch, aiming to alleviate the effect on psoriasis, within a randomized double-blind controlled trial [235]. The authors thought out the benefits of employing transdermal patches, which are similar to those of any other transdermal delivery system; however, the patches also provide better dose control. The most suitable substances for such delivery systems are lipophilic molecules with low molecular weight, and they also have lower melting points and higher volatility, characteristics associated with excellent skin penetration and relatively simple formulation [236]. Isaac and Holvey emphasized the impact of using transdermal patches in psychiatric medication, through which the legal concerns of the use of drugs with psychotropic action are overcome, preventing the possibility of drug abuse and facilitating the least restrictive affordable administration of prescribed medication as well. From an economic point of view, the cost of a transdermal formulation, particularly in the form of patches, has always been a subject of debate as the costs are much higher compared to the same treatment administered orally.

Nevertheless, the formulation of cannabinoids with the help of nanotechnology, along with many other drugs, is in continuous progress, allowing unique solutions to numerous challenges that would otherwise have remained unsolved. Future strategies in the field should provide at the same time accessible, reproducible, and cost-reduced solutions.

## 4. Conclusions

*Cannabis*-based products intended for administration on the skin should be developed by keeping the final target of the pharmacological action of compounds in mind. On one hand, those that target local actions in the upper layers of the skin can be formulated as conventional topical formulations (creams, gels, or ointments) by simply incorporating the compounds or natural extracts in the formulation’s base. On the other hand, for systemic actions, advanced transport systems that allow passage through all skin layers into systemic circulation should be developed.

Studies focusing on major dermatologic pathologies assess the potential of cannabinoids as a therapeutic alternative to conventional treatments, not only underlining their efficacy but also their good safety profile. Topical formulations containing natural cannabinoids have shown local analgesic, antipruritic, anti-inflammatory, cicatrizing, or antibacterial actions, demonstrating their benefits in the treatment of conditions such as psoriasis, atopic dermatitis, allergic contact dermatitis, eczema, lichen simplex, varicose ulcer, or epidermolysis bullosa. For millennia, the traditional use of natural cannabinoids has provided empirical evidence that they may have considerably fewer adverse events than current synthetic pharmacological agents. Because they are of natural origin, phytocannabinoids circumvent the drawbacks of synthetic compounds, which require significant resources both for production and for the removal of byproducts or solvents that are typically toxic or have undesirable effects. For these formulations, developing standardized products with an exact content of cannabinoids or standardized *Cannabis* extracts is required, and they should be clinically tested.

The treatment of conditions that involve the modulation of specific receptors, whether they are endocannabinoid receptors or other types of receptors, requires advanced processing to allow the systemic absorption of cannabinoids. In an attempt to develop such an advanced cannabinoid transport system, it would be ideal to consider the following aspects: (i) Only compounds of known purity can be used to prepare advanced delivery systems, while the active principle content from formulations based on plant extracts is difficult to optimize; (ii) the methods of loading active compounds need to be optimized to obtain precisely dosed products that release a well-defined quantity of the substance; (iii) controlled release systems that can maintain an effective plasma concentration as long as possible should be developed; (iv) cannabinoids exhibiting psychotropic effects should be preferably encapsulated in certain transport systems that prevent their passage through the BBB, and only peripheral CB receptors should be modulated; (v) production technologies have to be improved to circumvent excessively high costs that limit the access to such products.

## Figures and Tables

**Table 2:** Classification of the most important phytocannabinoids by their lipophilic nature.

Phytocannabinoid	LogP Octanol/Water	Chemical Structure
Cannabidiolic acid (CBDA)	4.86 [154]	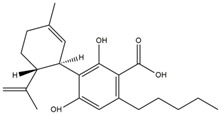
Cannabidiol (CBD)	5.79 [155]	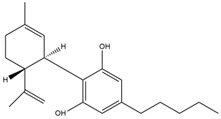
Cannabinodiol (CBND)	6.00 [156]	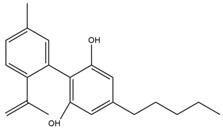
Δ9-trans-tetrahidrocannabinol (THC)	7.26 [157]	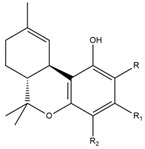
Δ8-trans-tetrahidrocannabinol (Δ8-THC)	7.4 [156]	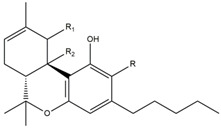
Cannabigerol (CBG)	7.47 [158]	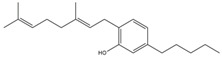
Cannabigerolic acid (CBGA)	8.31 [158]	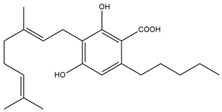
Tetrahydrocannabinolic acid (THCA)	8.41 [158]	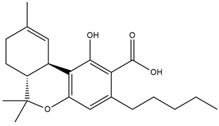

**Table 3:** Advanced formulations designed in order to penetrate the skin or membranes and to produce systemic effects.

Route of Absorption	Pharmaceutical Form Type	Disease	Observations	Reference
Transdermal	cannabinoids in a gel formulation using Transcutol^®^	increased penetration through the skin in various disease models	tested in vivo in two rat models of cocaine and alcohol addiction in the prevention of relapse to drug use	[179,180]
	ZYN001—Synthetic D-glyceryl acid ester prodrug of THC	fibromyalgia and neuropathic pain	tested by Zynerba Pharmaceuticals Inc. (USA), failed to achieve the desired blood levels of THC	[181]
	THC encapsulated in MLVs for transdermal applications.	pain treatment	tested in vitro for skin penetration by Altum Pharmaceuticals Inc. (USA)	[182]
	gel containing CBD using Transcutol^®^	epilepsy, developmental and epileptic encephalopathy, fragile-X syndrome, and osteoarthritis	developed and tested in vitro by Zynerba Pharmaceuticals Inc. (USA)	[183,184]
Transdermal	CBD and argan oil combination using Transcutol^®^ to encapsulate CBD	pain and inflammation associated with rheumatic or arthritic inflammatory disorders	Shemanky et al. [97] reported the beneficial effects of this formulation in a group of ten volunteer patients.	[185]
	THC prodrugs encapsulated using Transcutol^®^	patients with pathologically high intraocular pressure	tested by Zynerba Pharmaceuticals Inc.	[186,187]
Transdermal	stimulus-responsive chitosan/ZnO nanoparticles for transdermal absorption of CBD	epilepsy	delivery system was tested in vitro for drug release properties and cytotoxicity	[188]
	microemulsion containing THCA and CBDA	increased penetration through the skin in various disease models	tested in vitro for permeation improvement	[189]
	CBD emulsions stabilized with chitosan/collagen peptides nanoparticles	cosmetic applications	in vitro testing demonstrated that particle is able to penetrate the stratum corneum and diffuse into deeper layers of the skin	[190]
Transmucosal (oral)	oral formulation containing one or more cannabinoid–micelle encapsulation	Alzheimer’s disease, neuropathic pain, Dravet syndrome, Lennox–Gastaut syndrome, myoclonic seizures, juvenile myoclonic epilepsy, refractory epilepsy, schizophrenia, juvenile spasms, tuberous sclerosis complex, West syndrome, anxiety	not tested in vivo	[191]
	oral formulation containing one or more cannabinoids formulated as encapsulated micelles in the form of mucoadhesive gel, tablet, powder, liquid gel capsule, oral solution, granules, extrudates		tested in vivo on dogs by GW Research Limited (GB)	[192]
	oral formulation comprising a combination of at least two cannabinoids (THC or analogies and CBD or analogues)—in the form of mucoadhesive gel, tablet, powder, liquid gel capsule, oral solution, granules, extrudates	pediatric epilepsy	tested in vivo on dogs by GW Research Limited (GB)	[193]
	CBD or another cannabinoid conjugates	cancer	tested by Diverse Biotech Inc.	[194]
	TurboCBD™ delivery technology capsules	increasing circulating CBD in various disease	pharmacokinetic profile in a double-blinded, placebo-controlled, cross-over study on 12 healthy volunteers	[195]
	Canemes^®^—solid self-emulsifying pharmaceutical compositions comprising CBD and THC combination	pain-relieving drug	not tested in vivo	[196,197]
	Canemes^®^—cannabinoids chewing gum containing CBD/ CBDV/ THC/ CBC/ CBG combined with other compounds.	multiple sclerosis-related pain and spasticity, Parkinson’s disease, dementia, restless leg syndrome, and post-herpetic neuralgia	not tested in vivo	[198]
Transmucosal (oral)	Canemes^®^—CBD in the self-emulsifying delivery system	increases bioavailability in various disease	bioavailability tests in human	[199,200]
	Canemes^®^—cannabinoid derivatives and conjugates	acute and chronic pain	not tested in vivo	[201]
	Canemes^®^—CBD cyclodextrins; cannabinoids with sulfo-alkyl-β-CD	pain, Parkinson’s disease	clinical trial	[202,203]
	BRCX014—CBD with poloxamer 407, carboxymethyl cellulose, and starch	various disease	not tested in vivo	[204]
	BRCX014—CBD sublingual formulation	cancer treatment	not tested in vivo	[108,205]
	BCT-521—Combination of CBD and THC	pain management with cancer patients, Fibromyalgia, symptom relief in patients with advanced cancer	not tested in vivo	[205]
	ZYN001—synthetic D-glyceric acid ester prodrugTHC encapsulations in multilayered lipid vesicles	fibromyalgia and neuropathic pain pain treatment	not tested in vivo	[182]
Transmucosal (nasal)	nasal pharmaceutical product containing CBD plus other cannabinoids for topical application in the nasal cavity	schizophrenia	CBD+THC formulation tested on healthy volunteers	[206]
Transmucosal (pulmonary)	Canemes^®^—cannabinoid derivatives and conjugates	acute and chronic pain	not tested in vivo	[201]
	CBD mucoadhesive nanostructured lipid carriers	neuropathic pain	tested in vivo on mice model of neuropathic pain produced a better antinociceptive effect than oral or nasal administration of simple CBD oil	[207]
	Canemes^®^—cannabinoid derivatives and conjugates	acute and chronic pain	not tested in vivo	[201]
Trans-corneal	CBD-loaded mixed polymeric micelles of chitosan	degenerative and inflammatory disease of the eye	tested in vitro on human corneal epithelial cell line	[208]
Transmucosal (rectal)	CBD stable nanosized transfersomes as a rectal colloid	various diseases in which CBD has proven efficacy	tested in vivo for CBD release and nanoparticle delivery through the rectal membrane	[209]

Abbreviations: MLVs, multi-layered lipid vesicles; THC, tetrahydrocannabinol; CBD, cannabidiol; THCA, tetrahydrocannabinolic acid; CBDA, cannabidiolic acid; CBDV, cannabidivarin; CBC, cannabichromene; CBG, cannabigerol.

## Data Availability

No new data were created or analyzed in this study. Data sharing is not applicable to this article.

## Abbreviations

pCBs: phytocannabinoids; THC, tetrahydrocannabinol; Δ8-THC, Δ8-tetrahydrocannabinol; Δ9-THC, Δ9-tetrahydrocannabinol; CBD, cannabidiol; CBG, cannabigerol; CBN, cannabinol; CBE, cannabielsoin; CBC, cannabichromene; CBL, cannabicyclol; CBND, cannabinodiol; CBT, cannabitriol; ECS, endocannabinoid system; SC, stratum corneum; GPCRs, G-protein-coupled receptors; TRPV1, transient receptor potential vanilloid-1; TRPV3, transient receptor potential vanilloid-3; TRPA1, transient receptor potential ankyrin 1; PPARs, proliferator-activated receptors; AD, atopic dermatitis; ACD, allergic contact dermatitis; NB-UVB, narrowband ultraviolet B radiation; PUVA, psoralen Ultraviolet A radiation; TNF, tumor necrosis factor; IL, interleukin; UVB, ultraviolet B radiations; AEA, N-arachidonoyl ethanolamide; 2-AG, 2-arachidonoyl glycerol; PEA, N-palmitoyl ethanolamide, palmitoylethanolamide; ALEA, N-alpha-linolenoyl ethanolamide; LEA, N-linoleoyl ethanolamide; OEA, N-oleoyl ethanolamide; SEA, N-stearoyl ethanolamide; EPEA, N-eicosapentaenoyl ethanolamide; DHEA, N-docosahexaenoyl ethanolamide; TRP transient receptors; CCL8, C-C Motif Chemokine Ligand 8; CXCL10, C-X-C motif chemokine ligand 10; TH2, T helper 2; PPARγ, proliferative peroxisome-activated receptor gamma; IL, Interleukin; TNF-α, tumor necrosis factor α; HIF-1α, inducible factor-1α; VEGF, vascular endothelial growth factor; bFGF, basic fibro-blast growth factor; FAAH, fatty acid amide hydrolase enzyme; HDF, human dermal fibroblast; VEGF-A, vascular endothelial growth factor A; MMP-9, matrix metallopeptidase 9; CYP, cytochrome P450; AUC, area under the plasma concentration/time curve.
